# Previous TAVR in patients undergoing percutaneous edge-to-edge mitral valve repair (PMVR) affects improvement of MR

**DOI:** 10.1371/journal.pone.0205930

**Published:** 2018-10-19

**Authors:** Johannes Patzelt, Miriam Ulrich, Annika Becker, Karin A. L. Müller, Rezo Jorbenadze, Michal Droppa, Wenzhong Zhang, Sarah Mandel, Lisa Habel, Henning Lausberg, Janine Pöss, Tobias Geisler, Oliver Borst, Peter Rosenberger, Christian Schlensak, Meinrad Gawaz, Jürgen Schreieck, Peter Seizer, Harald F. Langer

**Affiliations:** 1 University Hospital, Department of Cardiology and Cardiovascular Medicine, Eberhard Karls University Tübingen, Tübingen, Germany; 2 Affiliated Hospital of Qingdao University, Department of Cardiology, Qingdao, Shandong, China; 3 University Hospital, Department of Cardiovascular Surgery, Eberhard Karls University Tübingen, Tübingen, Germany; 4 Medical Clinic II, Universitäres Herzzentrum Lübeck, University Hospital Schleswig-Holstein, Germany; 5 University Hospital, Department of Anesthesiology, Eberhard Karls University Tübingen, Tübingen, Germany; University of Mississippi Medical Center, UNITED STATES

## Abstract

**Background:**

Patients after transcatheter aortic valve replacement (TAVR) and persistent severe mitral regurgitation (MR) are increasingly treated with percutaneous edge-to-edge mitral valve repair (PMVR). The impact of a former TAVR on PMVR procedures is not clear.

**Methods and results:**

We retrospectively analyzed 332 patients undergoing PMVR using the MitraClip system with respect to procedural and clinical outcome. 21 of these 332 patients underwent TAVR before PMVR. Intra-procedural transthoracic (TTE) and transesophageal echocardiograms (TEE) immediately before and after clip implantation as well as invasive hemodynamic measurements were evaluated. At baseline, we found a significantly smaller mitral valve anterior-posterior diameter in the TAVR cohort (p < 0.001). A reduction of MR by at least three grades was achieved in a smaller fraction in the TAVR cohort as compared to the cohort with a native aortic valve (p = 0.02). Accordingly, we observed a smaller post-procedural cardiac output in the TAVR cohort (p = 0.02).

**Conclusion:**

PMVR in patients who had a TAVR before, is associated with altered MR anatomy before and a reduced improvement of MR after the procedure. Future larger and prospective studies will have to determine, whether a previous TAVR influences long-term clinical outcome of patients undergoing PMVR.

## Introduction

Patients with severe heart valve defects not eligible for conventional surgery can be treated with interventional techniques. For instance, patients with aortic stenosis and aortic valve regurgitation undergo transaortic valve replacement (TAVR) and patients with mitral regurgitation (MR) are treated with percutaneous mitral valve edge-to-edge repair (PMVR) using for example the MitraClip system. In a relevant fraction of these patients both aortic stenosis and MR are present. As a matter of fact, 20% to 30% of patients undergoing TAVR present with moderate to severe MR [1]. It has been reported that in up to two thirds of those patients, MR will improve after TAVR [1]. For the remainder of patients with residual severe MR, interventional treatment of the latter may be performed. There is insufficient data investigating, if the MitraClip procedure is influenced by the presence of a surgical or interventional aortic valve prosthesis.

With respect to PMVR, positive 5-year-outcome results were published recently, showing an increased rate of relapsing MR after one year compared to surgery, while beyond 1 year there was no difference compared to surgery [2], implying good stabilization of mitral ring geometry with PMVR. In line with these data, positive remodeling of the LV geometry with a reduction of the mitral valve anterior-posterior diameter immediately after the procedure was documented, which remained stable over time and was even more pronounced at follow-up [3]. To achieve this positive remodeling and to maintain permanent reduction of MR, the MitraClip needs to catch sufficient material of the anterior and posterior mitral valve leaflets, when the grasping maneuver is performed. This maneuver is directly influenced by the geometry of the mitral valve and of the heart [3, 4]. Interestingly, heart geometry is changed after TAVR. For example, LV mass index dropped by 25% at 3 years in the PARTNER cohort B trial [5]. Thus, the question arises, if those anatomic changes after TAVR have an impact on a later PMVR procedure.

This study was initiated to determine, whether PMVR using the MitraClip system in patients with a preceding TAVR is more difficult to carry out or is associated with inferior clinical outcome.

## Methods

### Study population

For this study, we retrospectively evaluated 339 consecutive patients, who underwent PMVR using the MitraClip system (Abbott Vascular, 3200 Lakeside Drive, Santa Clara, California, USA) between May 2014 and July 2017 at the University hospital, Department of Cardiology and Cardiovascular Medicine, University of Tübingen. Seven patients (5 with previous surgical mitral valve reconstruction, 1 with TAVR after surgical aortic valve replacement, 1 with surgical reconstruction of the aortic valve) were excluded from further analysis because of potential preexisting alterations. Of the remaining 332 patients in the study collective, 21 patients had undergone TAVR and 18 surgical aortic valve replacement (SAVR). The study was approved by the local ethics committee (Ethik-Kommission an der Medizinischen Fakultät der Eberhard-Karls-Universität und am Universitätsklinikum Tübingen, 260/2015R). The patients provided informed written consent to have their medical record data used in research. The decision for treatment by PMVR was made by an interdisciplinary heart team of interventional cardiologists and cardiac surgeons based on either the EuroSCORE [6] or on the presence of specific surgical risk factors not covered in the EuroSCORE. Exclusion criteria for PMVR were defined previously [4]. Heart failure patients had to be on optimal medical treatment according to current guidelines for at least 3 months prior to PMVR treatment [7].

### PMVR procedure

The procedure was carried out either in general anesthesia (GA) or in deep sedation (DS) as described before [4, 8, 9]. A right heart catheterization was performed at the beginning and at the end of the PMVR procedure, and cardiac output (CO) was determined according to the Fick method. TTE and TEE measurements were obtained in the hybrid operating room after induction of GA or DS, respectively, and at the end of the procedure. For echocardiography, we used Philips CX 50 or iE 33 machines (Philips HealthCare, Hamburg, Germany). All echocardiographic parameters were assessed at the beginning and at the end of the PMVR procedure. The severity of MR at baseline and the mechanism of regurgitation were determined as recommended in the current guidelines of the European Association of Echocardiography [10]. After PMVR, the residual MR was assessed according to the technique described by Foster et al. [11]. On the first postoperative day, venous blood samples were obtained from all patients for evaluation of complete blood count and levels of C-reactive protein (CRP). Medical records were used to identify complications during the hospital stay, procedure time and type of TAVR. Procedural failure was defined as the impossibility to implant a MitraClip^®^. Bleeding events were identified according to the VARC-2 criteria [12].

### Statistical analysis

Statistical analysis was performed with SPSS (version 24, IBM Deutschland GmbH, Ehningen, Germany). Categorical variables are displayed in percentages and absolute numbers. The level of significance in these variables was tested using the Chi-Square test. Ordinally scaled and continuous data are shown as mean ± standard deviation (SD). The Shapiro-Wilk test was used to check for normal distribution. In case of not normally distributed data, the Mann-Whitney-U test was used for inter-group comparisons. For normally distributed data the Student T test was performed. Two-tailed p values were calculated with a p-value < 0.05 being considered as statistically significant.

## Results

**Fig 1:**
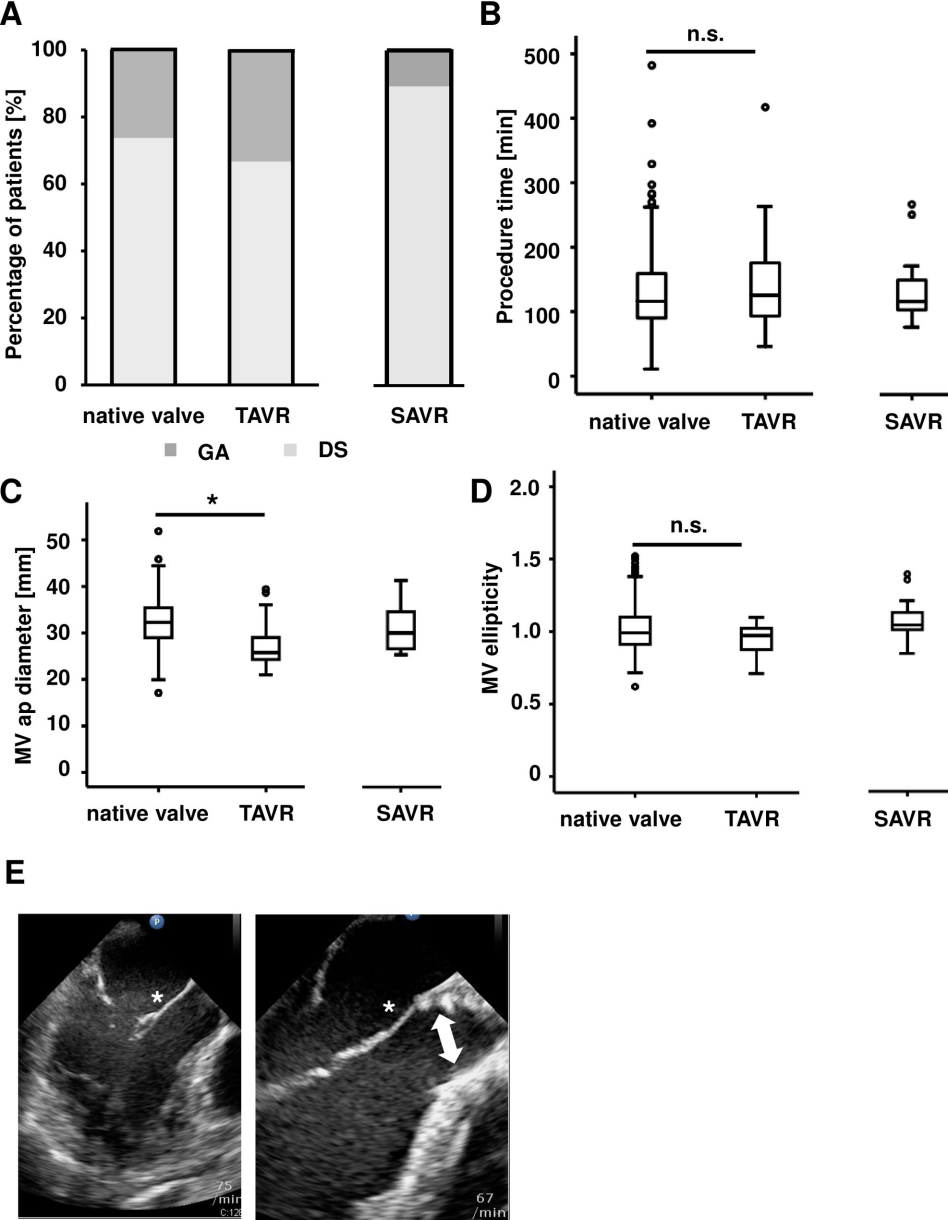
Procedural and echocardiographic characteristics before PMVR. The patient collective undergoing percutaneous edge-to-edge mitral valve repair (PMVR) was stratified into three cohorts according to a native aortic valve or a previous TAVR or SAVR procedure. A) Percentage of patients undergoing the PMVR procedure in deep sedation (DS) or in general anesthesia (GA). B) Procedure time for PMVR. Boxplots are depicting the median and the upper and lower quartile. C) The mitral valve anterior-posterior (ap) diameter is significantly smaller in patients with previous TAVR. Boxplots are depicting the median and the upper and lower quartile. D) The mitral valve ellipticity index was calculated at baseline. We observed no significant difference between patients with TAVR and without previous TAVR. Boxplots depict the median and the upper and lower quartile. E) Left panel: LVOT view in TEE demonstrating the anatomic situation in a patient with a native aortic valve (white asterisk indicates anterior mitral valve leaflet). Right panel: LVOT view in TEE demonstrating the anatomic situation in a patient after TAVR. Note the close relation of the distal part of the TAVR prothesis and the anterior mitral valve leaflet (white arrow indicates TAVR prosthesis).

In all patients receiving PMVR, we compared the cohort with a native aortic valve and the cohort with previous TAVR with regard to procedural and clinical parameters such as procedural success, reduction of MR and increase in cardiac output. The cohort with previous SAVR served as an additional point of orientation. In total, 332 patients were included in our study. Baseline characteristics are listed in **Table 1**. The mean age was 76.9 ± 8.9 years, and 57.2% of the patients were male. The etiology of MR was functional in 51.7%. 48.3% had a left ventricular ejection fraction (LVEF) of ≤ 35% and the mean EuroSCORE II was 10.8 ± 10.3. There were significant differences regarding the baseline characteristics between the cohort with a native aortic valve and the cohort with previous TAVR. A significant smaller fraction of patients in the cohort with a native aortic valve had coronary heart disease (71.0% vs. 95.2% in the TAVR cohort, p = 0.02) and also hyperlipoproteinemia was significantly decreased (44.0% vs. 66.7%, p = 0.04). In the cohort with previous TAVR, a higher fraction of patients had renal insufficiency as compared to the cohort with a native aortic valve (71.4% vs. 46.8%, p = 0.03). EuroSCORE II levels were higher in the TAVR cohort (11.9 ± 6.4 vs. 10.8 ±10.3 in the group with a native aortic valve, p = 0.02). Less patients in the TAVR cohort were treated with betablockers (71.4% vs. 89.1% in the cohort with a native aortic valve, p = 0.02) and with ACE inhibitors/sartans (66.7% vs. 84.8%, p = 0.03). Besides that, the TAVR and the native aortic valve group were balanced and comparable regarding the baseline characteristics. 73.7% of patients with native aortic valve and 66.6% of patients with TAVR underwent the PMVR procedure in deep sedation (**Table 1, Fig 1A**). As a point of orientation, we also looked at the cohort with SAVR in which 88.9% had deep sedation (**Table 1, Fig 1A**). Although the absolute numbers were different, there was no significant difference with respect to procedure time between the cohorts (130 ± 60 min in the cohort with a native aortic valve, 145 ± 83 min in the cohort with TAVR; p = 0.52 vs. the cohort with a native aortic valve; 133 ± 53 min with SAVR; **Fig 1B**). At baseline, we observed a significantly smaller mitral valve anterior-posterior diameter in the TAVR cohort. The diameter was 32 ± 5 mm in the cohort with a native aortic valve vs 28 ± 5 mm in the cohort with previous a TAVR (p < 0.001). In the cohort with SAVR, the anterior-posterior diameter was 31± 5 mm (**Fig 1C**). There were no significant differences between the cohorts regarding mitral valve ellipticity, defined as anterior-posterior annulus diameter divided by the medial-lateral diameter (1.01 ± 0.15 in the native aortic valve cohort vs. 0.94 ± 0.11 in the TAVR cohort (p = 0.11) and 1.07 ± 0.14 in the SAVR cohort (**Fig 1D**). **Fig 1E** demonstrates the anatomic situation of the mitral valve with a native aortic valve (left panel) and with a TAVR prosthesis (right panel).

**Table 1:** Patient baseline characteristics.

	complete collective n = 332	native aortic valve n = 293	TAVR n = 21	P–value	SAVR n = 18
Age	76.9 (±8.9) (332/332)	76.7 (±8.9) (293/293)	79.2 (±10.2) (21/21)	0.05	76.6 (±6.3) (18/18)
Male gender	57.2% (190/332)	56.0% (164/293)	66.7% (14/21)	0.34	66.7% (12/18)
Coronary heart disease	73.2% (243/332)	71.0% (208/293)	95.2% (20/21)	0.02	83.3% (15/18)
Atrial fibrillation	65.7% 218/332	67.6% (198/293)	57.1% (12/21)	0.33	44.4% 8/18
Hypertension	70.5% (234/332)	70.0% (205/293)	85.7% (18/21)	0.12	61.1% (11/18)
Smoker	17.2% 57/332	16.7% (49/293)	23.8% (5/21)	0.41	16.7% (3/18)
Hyperlipoproteinemia	45.5% (151/332)	44.0% (129/293)	66.7% (14/21)	0.04	44.4% (8/18)
Diabetes	28.6% (95/332)	28.3% (83/293)	42.9% 9/21	0.16	16.7% (3/18)
NYHA-class	3.2 (2 to 4) (325/332)	3.2 (2 to 4) (286/293)	3.4 (2 to 4) (21/21)	0.11	3.1 (2 to 4) (18/18)
*Renal insufficiency	47.9% (159/332)	46.8% (137/293)	71.4% (15/21)	0.03	38.9% (7/18)
*Pulmonary hypertension	65.2% (214/328)	64.4% (186/289)	76.2% (16/21)	0.27	66.7% (12/18)
Euroscore II	10.8 ± 10.3 (328/332)	10.5 ± 10.4) (289/293)	11.9 ± 6.4 (21/21)	0.02	15.7 ± 11.4 (18/18)
LVEDD	54.0 ± 10.0 (292/332)	54.4 ± 9.9 (257/293)	53.6 ± 9.2 (19/21)	1.0	50.5 ± 12.2 (16/18)
LV Function					
≤35%	48.3% (160/331)	49.7% (145/292)	33.3% 7/21	0.15	44.4% (8/18)
36–50%	26.0% (86/331)	22.9% 67/292	52.4% (11/21)	0.003	44.4% (8/18)
>50%	25.7% (85/331)	27.4% (80/292)	14.3% (3/21)	0.19	11.1% (2/18)
Etiology of MR					
Functional	51.7% (169/327)	52.4% (151/288)	42.9% 9/21	0.69	50% (9/18)
Degenerative	48.3% 158/327	47.6% 137/288	57.1% 12/21	0.69	50% (9/18)
Betablockers	87.6% 282/322	89.1% 253/284	71.4% (15/21)	0.02	82.4% (14/17)
Aldosteronantagonist	51.3% (164/320)	53.5% (152/282)	38.1% (8/21)	0.16	23.5% (4/17)
ACE inhibitors/sartans	83.8% (268/320)	84.8% (239/282)	66.7% (14/21)	0.03	88.2% (15/17)
Diuretics	88.5% (284/321)	88.7% (251/283)	85.7% (18/21)	0.68	88.2% (15/17)
Digitalis	10.3% 33/320	11.7% (33/282)	0.0% (0/21)	0.10	0.0% (0/17)
Calcium antagonists	18.8% (60/319)	18.1% (51/281)	28.6% (6/21)	0.24	17.6% (3/17)
Anticoagulation	68.7% (222/323)	70.2% (200/285)	52.4% (11/21)	0.09	64.7% (11/17)
General anesthesia	25.9% (86/332)	26.3% (77/293)	33.3% (7/21)	0.48	11.1% (2/18)
Deep sedation	74.1% (246/332)	73.7% (216/293)	66.6% (14/21)	0.48	88.9% 16/18
No. of implanted clips					
0 (failure)	1.8% (6/332)	1.4% (4/293)	4.8% (1/21)	0.23	5.6% (1/18)
1	49.7% 165/332	48.1% (141/293)	61.9% (13/21)	0.23	61.1% (11/18)
2	40.1% (133/332)	41.3% (121/293)	33.3% (7/21)	0.47	27.8% (5/18)
3	7.8% (26/332)	8.5% 25/293	0.0% (0/21)	0.16	5.6% (1/18)
4	0.3% (1/332)	0.3% (1/293)	0.0% (0/21)	0.79	0.0% (0/18)
5	0.3% (1/332)	0.3% (1/293)	0.0% (0/21)	0.79	0.0% (0/18)

ACE = angiotensin converting enzyme, LV = left ventricular, LVEDD = left ventricular enddiastolic diameter

* as defined for Euroscore II

**Fig 2:**
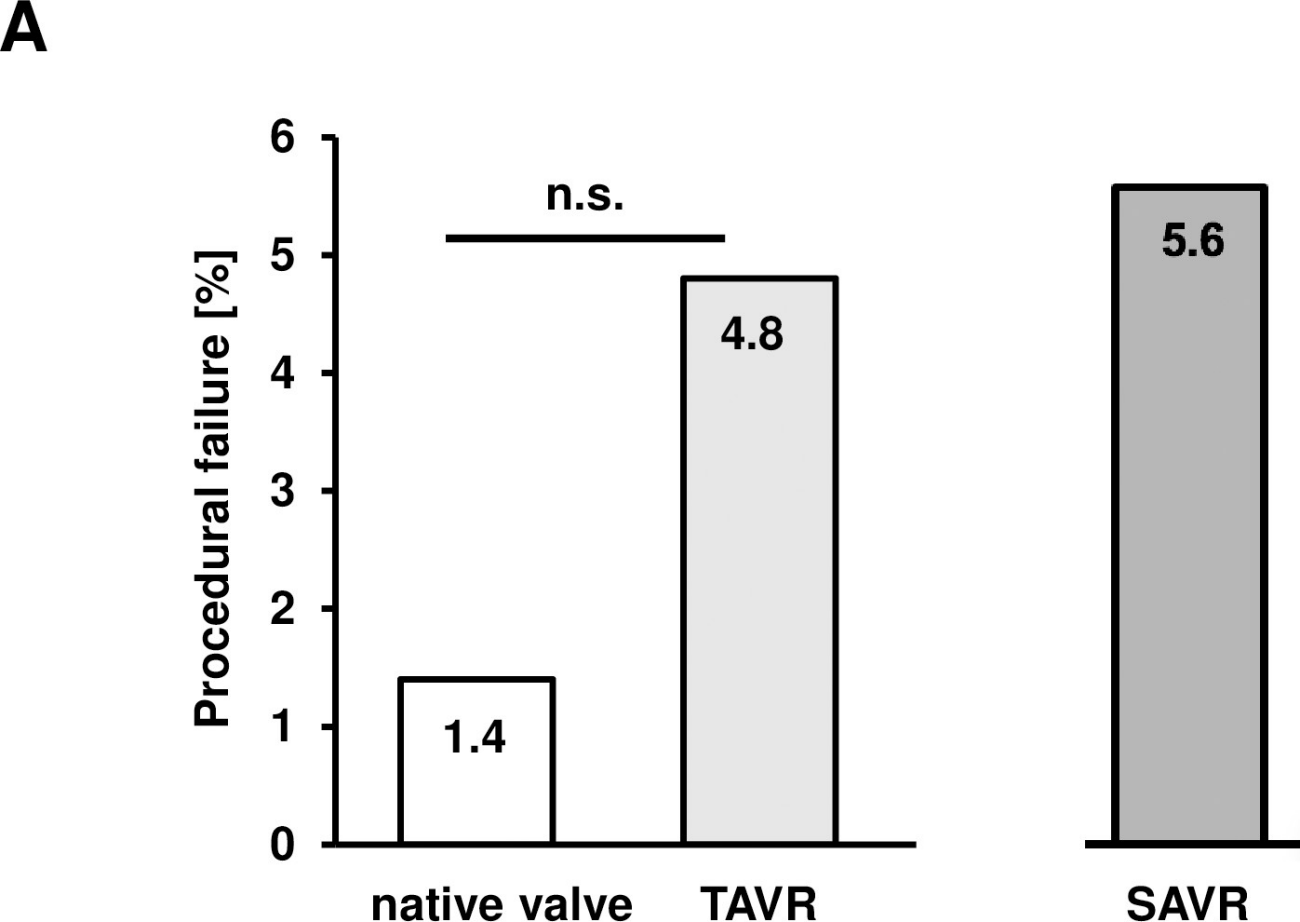
Procedural success of the PMVR procedure. A) Comparison of procedural failure. Procedural failure was 1.4% in the native aortic valve cohort vs 4.8% in the TAVR cohort (p = 0.23 vs. the cohort of patients with a native aortic valve) and 5.6% in the SAVR cohort.

Comparing the procedural outcome data, we found no significant difference between the cohorts with respect to procedural failure. Procedural failure was 1.4% in the native aortic valve cohort vs. 4.8% in the TAVR cohort (p = 0.23 vs. the cohort with a native aortic valve) and 5.6% in the SAVR cohort (**Fig 2A**). In **Table 2**, the implanted TAVR models are listed and the corresponding success rates of the PMVR procedures are given. Procedural success rates were 8/9 in patients with a CoreValve, (Medtronic, 710 Medtronic Parkway, Minneapolis, Minnesota 55432–5604, USA), 9/9 in patients with an Edwards Sapien (Edwards Lifesciences Corp., One Edwards Way Irvine, CA 92614, USA), 2/2 in patients with a Lotus (Boston Scientific, 300 Boston Scientific Way, Marlborough, MA 01752–1234, USA), and 1/1 in patients with a Symetis (Boston Scientific, 300 Boston Scientific Way, Marlborough, MA 01752–1234, USA) prosthesis. Detailed data on the different types and sizes of TAVR prosthesis are given in **S1 Table**.

**Table 2:** Types of implanted TAVR devices.

Device type	No. of patients	Success in PMVR
CoreValve	9	8
Edwards sapien	9	9
Lotus	2	2
Symetis	1	1

**Fig 3:**
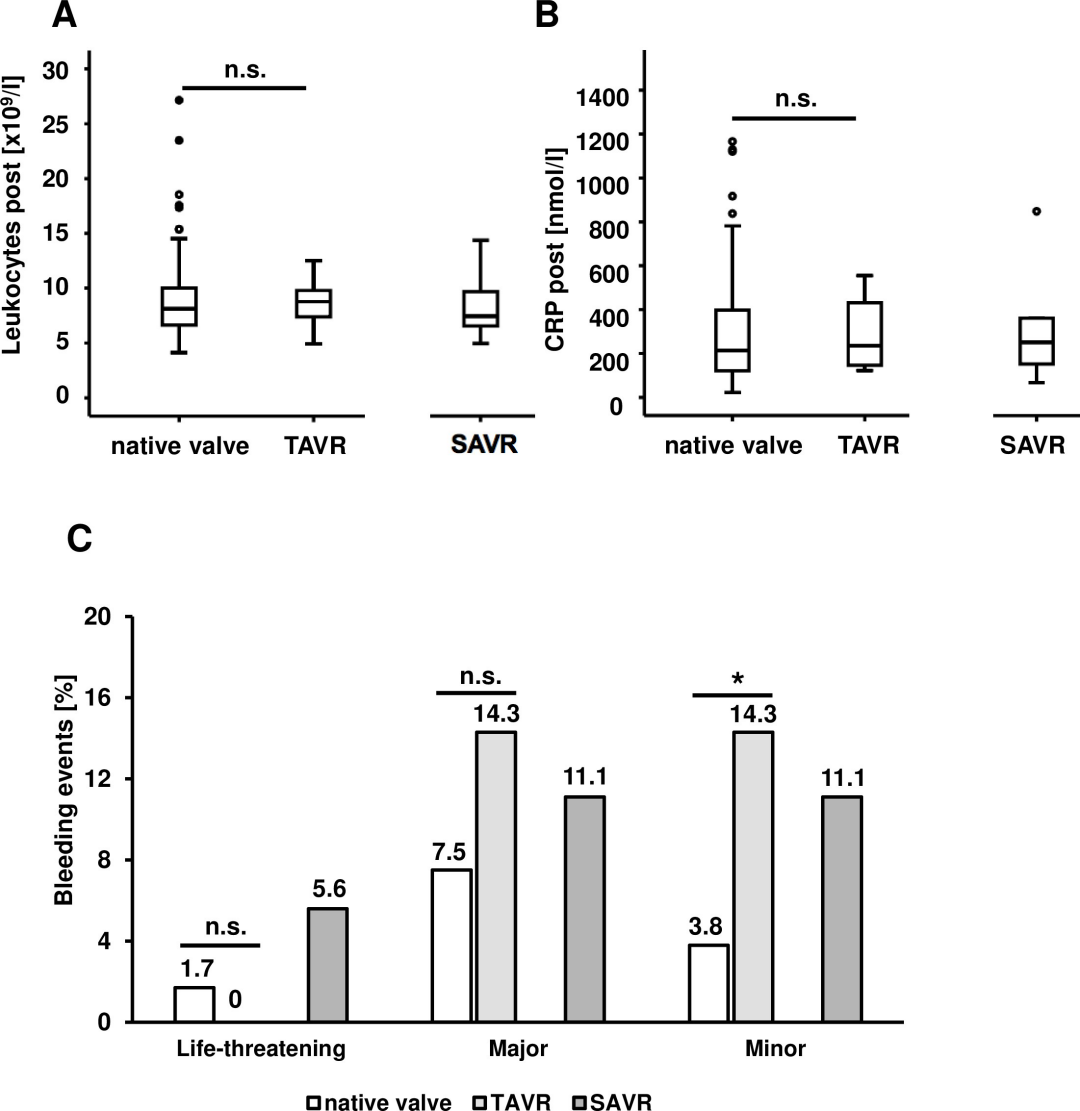
Procedural complications. A) There was no significant difference in post-interventional leukocyte count in patients with a TAVR prosthesis and those without. Boxplots depict the median and the upper and lower quartile. B) There was no significant difference in post-interventional CRP levels in patients with a TAVR prosthesis and those without. Boxplots show the median and the upper and lower quartile. C) No difference between the TAVR and the control cohorts could be detected with regard to life-threatening and major bleedings, while there were significantly more minor bleedings in the TAVR cohort (p = 0.02).

When we evaluated postoperative inflammation, we could not detect a significant difference between the cohorts. Postinterventional leukocyte counts were 8.6 ± 3.0 x 10^**9**^/l in the native aortic valve cohort, 8.8 ± 2.2 x 10^**9**^/l in the TAVR cohort (p = 0.45 vs. the cohort with a native aortic valve) and 8.1 ± 2.6 x 10^**9**^/l in the SAVR cohort (**Fig 3A**). Postinterventional CRP levels were 310 ± 298 nmol/l in the native aortic valve cohort, 285 ± 158 nmol/l in the TAVR cohort (p = 0.52 vs. the cohort with a native aortic valve), and 386 ± 384 nmol/l in the SAVR cohort (**Fig 3B**). Postinterventional bleeding complications were also evaluated. As defined by the VARC-2 (Valve Academic Research Consortium) criteria [12], those were stratified in life-threatening, major, or minor bleeding. Reasons for postinterventional bleeding were access site bleeding, urogenital and gastrointestinal bleeding, endobronchial bleeding, or bleeding from the injection site of the central venous catheter. With respect to life threatening bleeding there was no significant difference between the cohorts: Respective bleeding rates were 1.7% in the cohort with a native aortic valve, 0.0% in the TAVR cohort (p = 0.55 vs. the cohort with a native aortic valve) and 5.6% in the SAVR cohort. Major bleeding rates were 7.5% in the native aortic valve cohort, 14.3% in the TAVR cohort (p = 0.27 vs. the cohort with a native aortic valve), and 11.1% in the SAVR cohort. Minor bleeding rates were 3.8% in the cohort with a native aortic valve, 14.3% in the TAVR cohort (p = 0.02 vs. the cohort with a native aortic valve) and 11.1% in the SAVR cohort. All bleeding rates are depicted in **Fig 3C**.

**Fig 4:**
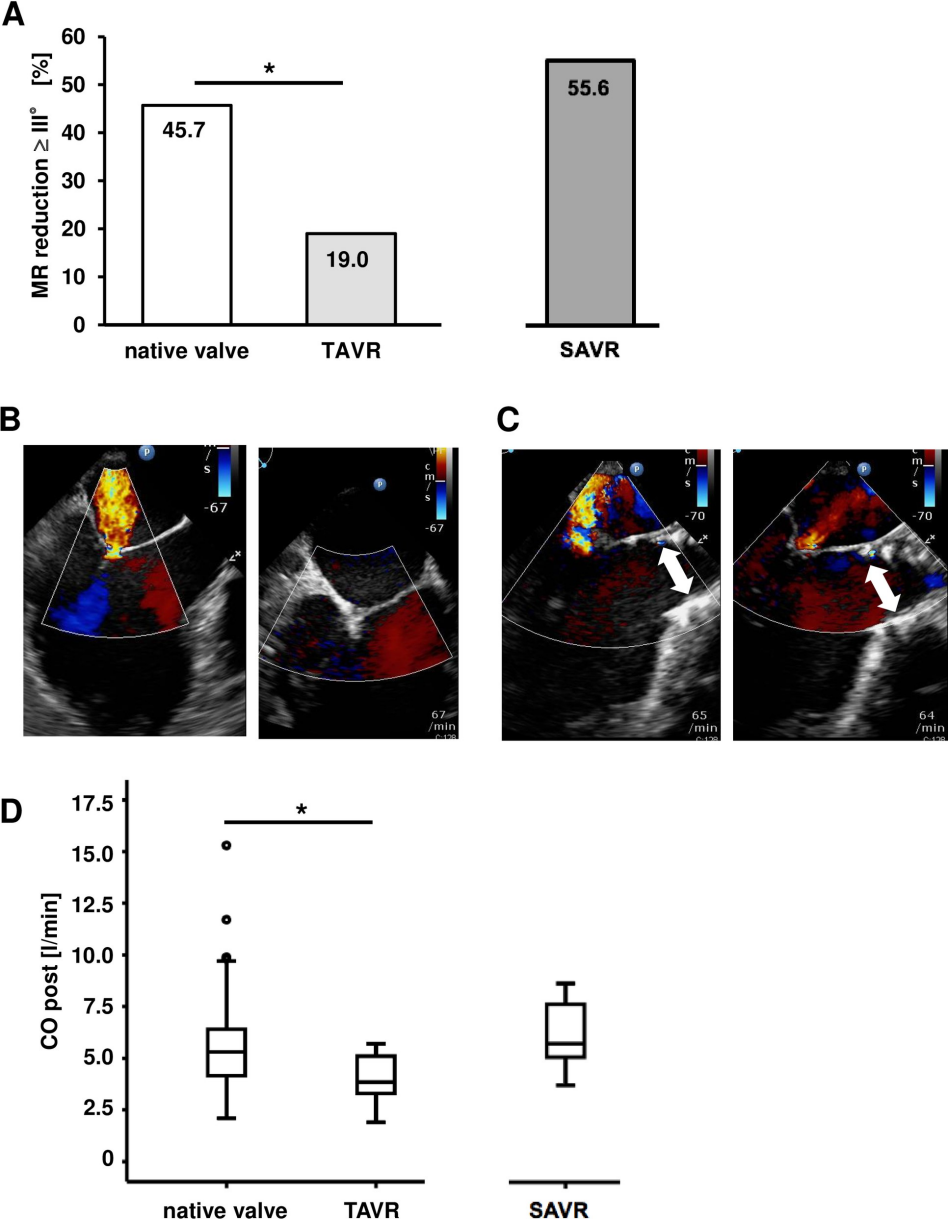
Procedural outcomes. A) In the cohort of patients with a native aortic valve a reduction of MR of at least 3 grades was achieved in 45.7% of patients as compared to 19.0% of patients in the cohort with TAVR (p = 0.02 vs. the cohort of patients with a native aortic valve) and 55.6% in the cohort with SAVR. B) Color flow doppler imaging (LVOT view in TEE) of the MR before (left panel) and after (right panel) the PMVR procedure in a patient with a native aortic valve. C) Color flow doppler imaging (LVOT view in TEE) of the MR before (left panel) and after (right panel) the PMVR procedure in a patient with a TAVR prothesis (white arrow indicates the TAVR prosthesis). D) Cardiac output after PMVR was 5.5 ± 1.9 l/min in the cohort with a native aortic valve, 4.1 ± 1.2 l/min in the cohort with TAVR (p = 0.01 vs. the cohort of patients with a native aortic valve) and 6.2 ± 1.6 l/min in the cohort with SAVR.

Interestingly, the achieved reduction of MR by PMVR was less in the cohort with previous TAVR as compared to the cohort of patients with a native aortic valve. In the cohort with a native aortic valve, a reduction of MR of at least 3 grades was achieved in 45.7% of patients compared to 19.0% of patients in the cohort with TAVR (p = 0.02 vs. the cohort of patients with a native aortic valve) and 55.6% in the cohort with SAVR (**Fig 4A**). For better illustration of the intraprocedural situation, **Fig 4B** depicts a TEE image (left ventricular outflow tract view) of MR before and after PMVR in a patient with a native aortic valve, **Fig 4C** of a patient with a TAVR prosthesis. Furthermore, the cardiac output after PMVR was significantly lower in the cohort with TAVR as compared to the control cohorts. Cardiac output after PMVR was 5.5 ± 1.9 l/min in the cohort with native aortic valve, 4.1 ± 1.2 l/min in the cohort with TAVR (p = 0.01 vs. the cohort with a native aortic valve), and 6.2 ± 1.6 l/min in the cohort with SAVR (**Fig 4D**). At baseline, there was no significant difference with respect to cardiac output between the three cohorts. At baseline, cardiac output was 4.7 ± 1.6 l/min in the cohort of patients with a native aortic valve, 4.2 ± 0.7 l/min in the cohort with TAVR (p = 0.55 vs. the cohort with a native aortic valve), and 4.8 ± 1.4 l/min in the cohort with SAVR (**S1 Fig**).

## Discussion

The impact of a pre-existing TAVR prosthesis in patients undergoing a PMVR procedure is not clear. Here, we describe in a large patient collective that i) mitral valve anatomy in patients with previous TAVR is altered, ii) the percentage of procedural success regarding MR is significantly lower and iii) that cardiac output is significantly reduced after PMVR in patients who have previously undergone a TAVR procedure.

PMVR is a successful and increasingly performed treatment option for patients with MR who are not eligible for conventional surgery [13]. Both TAVR and PMVR are commonly used in patients of older age, high perioperative risk—as assessed by the EUROSCORE [14, 15] or the STS score [15, 16]—and severe comorbidities not included in these scores. Recent data from the European MitraClip registry report that 8.6% of patients receive both aortic valve repair (of those 68.4% SAVR and 31.6% TAVR) and PMVR [17]. TAVR is an accepted therapy for aortic stenosis in patients not eligible for conventional surgery [18], but it also brings along negative side effects such as the need for pace maker implantation. Depending on the type of prosthesis, 8.5–25.9% of patients undergoing TAVR require a new permanent pacemaker (PPM) within 30 days after the procedure [19–23]. Furthermore, the implantation technique influences the rate of pace maker-dependency after TAVR [24]. This observation can be explained by specific effects on the heart valve apparatus and adjunct structures such as the AV-node [24]. In line with this, interesting observations were made, for example that LV mass index dropped by 25% at 3 years after TAVR in the PARTNER cohort B trial [5]. Moreover, TAVR may directly influence the anatomy of the mitral valve, which could be attributable to a directed force of a TAVR prosthesis onto the mitral valve apparatus. In line with this hypothesis, we found a significantly smaller mitral valve anterior-posterior diameter at baseline in the group with TAVR in comparison to the group with a native aortic valve. Interestingly, SAVR does not seem to impact on the mitral valve annulus in the same way: The mitral valve annulus anterior-posterior diameter in patients with SAVR did not differ significantly from that in patients with native aortic valve.

In the literature, mitral valve stenosis was observed in some cases after TAVR [25, 26]. Interestingly, both reported cases refer to a CoreValve prosthesis. According to our impression, implantation of a CoreValve prosthesis may have an impact on the movement of the anterior mitral valve leaflet, if the prosthesis is deployed rather low in the LVOT. One may speculate, that the impaired movement of the anterior mitral leaflet in patients with previous TAVR could hamper the grasping of the mitral valve leaflets during PMVR. In line with this, the one procedural failure we observed in the TAVR group occurred in a patient with a CoreValve prosthesis. However, the patient cohort with TAVR was more severely diseased with a significant larger fraction of patients with coronary heart disease compared to the native aortic valve cohort (95.2% vs. 71.0%; p = 0.02), hyperlipoproteinemia (66.7% vs. 44.0%, p = 0.04) and renal insufficiency (71.4% vs. 46.8%; p = 0.03). Consequently, EuroScore II levels were significantly higher in the cohort with TAVR (11.9 ± 6.4 vs. 10.5 ± 10.4; p = 0.02). Thus, we cannot rule out that those confounding factors are at least in part responsible for the reduced procedural success. We are aware that with this rather small patient collective, it can be no more than an observation that the device and its implantation technique had an impact on the success of the PMVR procedure, but this hypothesis should be tested in larger trials.

PMVR using the MitraClip system is influencing the mitral valve apparatus, too. As we could recently demonstrate, the procedure results in a reduction of mitral annular size, which correlates inversely with residual MR and is stable at follow up [3]. These observations indicate that both PMVR and TAVR profoundly change heart geometry and may affect implantation success, if the one or the other is present. Interestingly, there was no difference in life-threatening and major bleedings between the two groups, while there were significantly more minor bleedings in the group with a previous TAVR procedure. One possible explanation of higher bleeding rates might be that significantly more patients in the group with previous TAVR had coronary heart disease (95.2% vs. 71% in the group with native aortic valve; p = 0.02) and, thus, presumably had more frequently an antiplatelet therapy.

Besides affecting mitral valve geometry, PMVR has short-term and long-term effects on hemodynamics. For instance, we found an immediate increase of cardiac output after PMVR from 4.6 ± 1.4 l/min to 5.4 ± 1.6 l/min (p < 0.001) [8]. Gaemperli and colleagues could demonstrate in a study using measurements with a conductance catheter that hemodynamic profiles were improved with a reduction of left ventricular preload while left ventricular contractility was preserved [27]. In the recently published 5 year-results of the Everest II study, Feldman et al. describe a stable reduction of left ventricular end-diastolic and end-systolic volumes at 5 year-follow-up [2]. If not heavily calcified, the mitral ring is quite flexible. In line with this, we observed in a preceding study a reduction in mitral annulus diameter and improved leaflet coaptation in patients ventilated with elevated PEEP [4]. Interestingly, in the presence of a TAVR prosthesis, MR reduction and CO post PMVR were significantly lower. At present, we cannot finally explain this observation, but the more complex heart geometry in these patients may have partially contributed to this clinical result. However, we cannot entirely rule out other confounding factors, which will have to be determined in larger trials and basic research studies addressing this phenomenon. Our study adds further important aspects to the discussion of how to treat patients with complex valvular heart disease not eligible for conventional surgery. Nevertheless, future prospective studies are needed to define the optimal protocol for an interventional approach in patients suffering from both severe aortic stenosis and mitral regurgitation.

### Study limitations

This study has clear limitations. One of the main limitations is the limited sample size, particularly of patients, who had both a previous TAVR prosthesis and the indication for PMVR in our retrospective collective. There were some imbalances between the cohort of patients with a native aortic valve and that with TAVR. Due to the limited sample size of the TAVR cohort, an adjusted analysis was not performed. Moreover, different device types of TAVR prostheses were implanted in this study patient collective, which may differ in their influence on heart geometry [28]. Similarly, different access routes of former TAVR implantation (trans-femoral, trans-subclavian, trans-apical) might influence results of PMVR. Due to the small size of the TAVR cohort, adjustment for device type and access route was not carried out. Clearly, the event rate of procedural failure was too small to draw any conclusions in that respect. Nevertheless, our data represent a real life all comer collective. Given the scarce data on this specific constellation and the complexity of the procedure, the patient number appeared reasonable to generate hypotheses, which will have to be scrutinized in future trials.

## Conclusions

Here, we assessed whether the presence of a TAVR prosthesis in patients undergoing additionally PMVR therapy using the MitraClip system has any clinical relevance. Interestingly, we observed altered mitral valve annulus diameter geometry depending on the presence of a TAVR prosthesis, and patients, who had previous TAVR treatment showed less improvement of MR and a smaller CO after PMVR. Future analyses on larger patient collectives and prospective trials have to further scrutinize the question, how outcome of PMVR is influenced by a previous TAVR procedure.

## Supporting information

S1 Table Types of implanted TAVR prostheses. (DOC)

S1 Fig Cardiac output at baseline. At baseline, cardiac output was 4.7 ± 1.6 l/min in the cohort with a native aortic valve, 4.2 ± 0.7 l/min in the cohort with TAVR (p = 0.55 vs. the cohort of patients with a native aortic valve), and 4.8 ± 1.4 l/min in the cohort with SAVR.
(TIF)

S1 File Original data set. (XLSX)
