# Supplementary material for: Previous TAVR in patients undergoing percutaneous edge-to-edge mitral valve repair (PMVR) affects improvement of MR
Source: PLoS One. 2018 Oct 19;13(10):e0205930. doi: 10.1371/journal.pone.0205930 (PMC6195292; doi:10.1371/journal.pone.0205930)
Supplement: S1 Table — (DOC) [file pone.0205930.s001.doc]

**Supplemental Table 1: Types of implanted TAVR prostheses**

|  | CoreValve | Edwards Sapien | Lotus | Symetis |
| --- | --- | --- | --- | --- |
| TAVR type (no. of patients) | CoreValve 26 mm (7)  CoreValve 29 mm (1)  Corevalve evolute 29 mm (1) | Edwards sapien 23 mm (1)  Edwards sapien 26 mm (3)  Edwards sapien 3 26 mm (2)  Edwards sapien 3 29 mm (3) | Lotus valve 25 mm (2) | Symetis L (1) |
