# Supplementary figures and images for: Previous TAVR in patients undergoing percutaneous edge-to-edge mitral valve repair (PMVR) affects improvement of MR
Source: PLoS One. 2018 Oct 19;13(10):e0205930. doi: 10.1371/journal.pone.0205930 (PMC6195292; doi:10.1371/journal.pone.0205930)

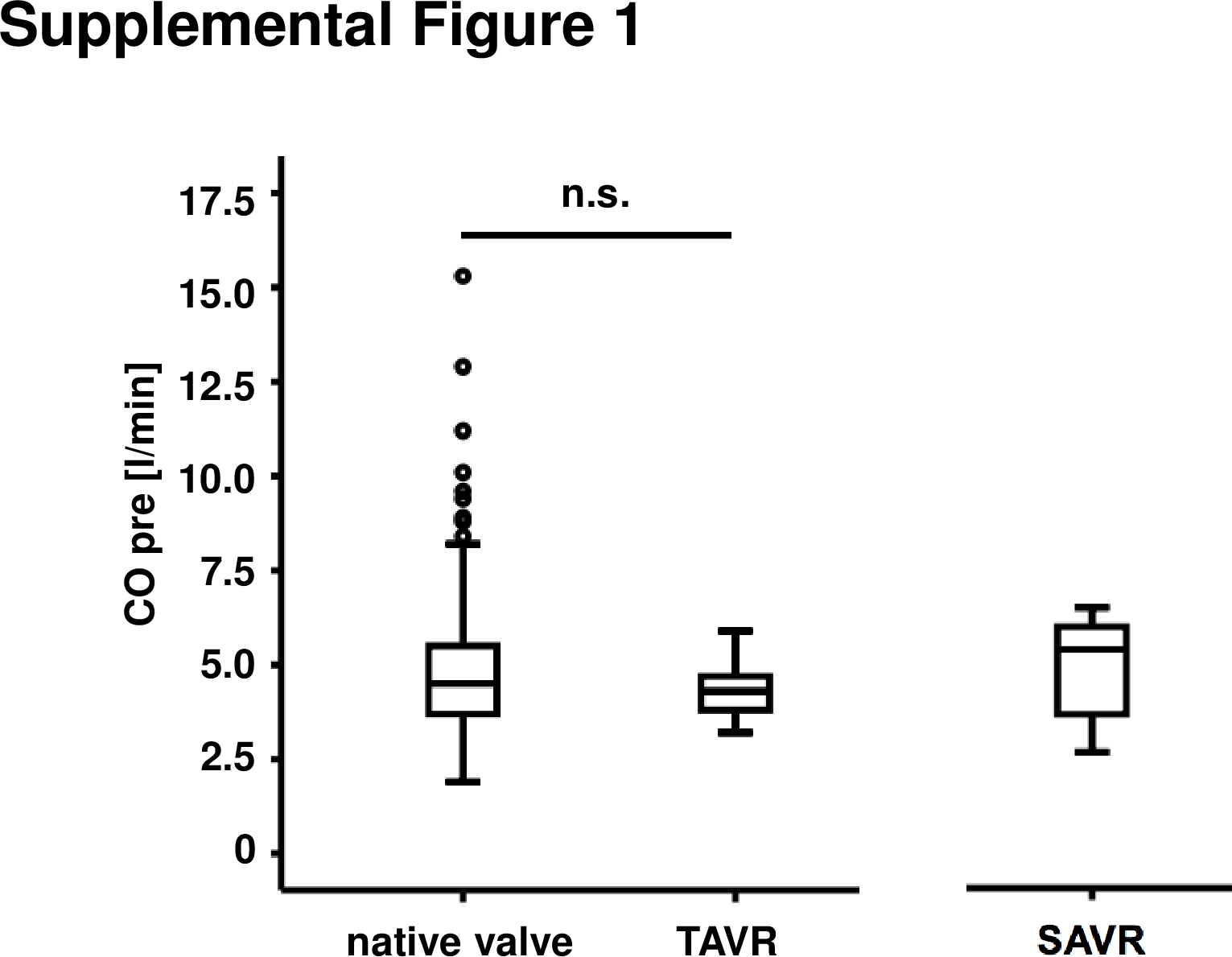

Supplement: S1 Fig — At baseline, cardiac output was 4.7 ± 1.6 l/min in the cohort with a native aortic valve, 4.2 ± 0.7 l/min in the cohort with TAVR (p = 0.55 vs. the cohort of patients with a native aortic valve), and 4.8 ± 1.4 l/min in the cohort with SAVR. (TIF) [file pone.0205930.s002.tif]
